# Draft genome sequence of *Lactobacillus rhamnosus C25*, isolated from an Indian dairy cheese

**DOI:** 10.1128/mra.00534-24

**Published:** 2025-02-13

**Authors:** Daraksha Iram, Manish Singh Sansi, Shilpa Vij

**Affiliations:** 1 Antimicrobial Peptides, Biofunctional Probiotics & Peptidomics Laboratory, Dairy Microbiology Division, National Dairy Research Institute, Karnal, Haryana, India; 2 Biofunctional Peptidomics & Metabolic Syndrome Laboratory, Animal Biochemistry Division, National Dairy Research Institute, Karnal, Haryana, India; The University of Arizona, Tucson, Arizona, USA

**Keywords:** *Lactobacillus*, WGS, genome annotation

## Abstract

We announce here the draft genome sequence of *Lacticaseibacillus rhamnosus C25*, a lactic acid bacterium participating in fermentation processes and having probiotic potential, isolated from Cheddar cheese. The *C25* genome is 2.97 Mb in size, comprising 2,885 gene sequences assembled into 62 scaffolds.

## ANNOUNCEMENT

The human gut microbiota, particularly the genus *Lactobacillus*, plays a vital role in metabolism, pathogen control, and immune regulation. Found in the gastrointestinal and urogenital tracts, oral cavity, and breast milk, *Lactobacillus* demonstrates strong probiotic properties and adapts to diverse environments, including dairy and non-dairy sources (1–4). Certain strains serve as probiotics or starter cultures in food fermentation, showing resilience to acidity and bile and enabling temporary colonization of the gut. Consuming *Lactobacillus* has been linked to improved gut homeostasis and reduced symptoms of irritable bowel syndrome (IBS) (5–7). The C25 strain, isolated from 6-month-ripened Cheddar cheese from the experimental dairy plant, National Dairy Research Institute, India, was preserved as a glycerol stock at −80°C following Succi et al. (2005) (8). A single colony from de Man, Rogosa, and Sharpe Agar/Broth (MRS) agar was cultured in MRS broth at 37°C with shaking, harvested by centrifugation, and stored at −20°C for genomic DNA isolation. DNA extraction and 16S rRNA gene amplification using universal primers followed Heilig (2002) (9) (GenBank accession KF806538). The *C25* culture was incubated for 16–20 h at 37°C in MRS-media (Himedia, Mumbai, India), followed by genomic DNA isolation via AlexGen bacterial gDNA extraction kit (Alexius Biosciences, Germany) protocol. DNA was prepared for sequencing using a QIAseq FX DNA Library UDI-B Kit (QIAGEN, Hilden, Germany), with modifications including 5 ng of DNA input and elongation time extended to 1 min during PCR. Sequencing was conducted on the Illumina Novaseq 6000 platform with a 391 bp paired-end-read protocol. High-fidelity amplification utilized HiFi PCR Master Mix, followed by analysis on the TapeStation 4150 (Agilent Technologies) with high-sensitivity D1000 ScreenTape. Genome libraries, comprising 12,370,872 reads with total base pair values of 1,966,968,648 bp were obtained for *C25. De novo* assembly was performed using KmerGenie (10), Velvet (11), SPAdes (12), and GapFiller (13), resulting in 62 scaffolds with an N50 of 129,392 bp and a maximum scaffold size of 311,581 bp, covering a genome size of 2.97 Mb. Default parameters were used for all software. It provided insights into the relative genome length compared with other sequenced *L. rhamnosus* strains (14, 15). Annotation was added by the National Center for Biotechnology Information (NCBI) Prokaryotic Genome Annotation Pipeline (PGAP) (Li et al., 2021) (16). Genome exhibited a completeness of 98.65% by CheckM analysis (v1.2.2) and a GC content of 46.5%. The C25 genome comprised 2,885 genes, including 2,821 CDSs, 2,746 protein-coding genes, 55 tRNA genes, and 6 rRNA genes (three 5S, two 23S, and one 16S). BlastP annotation against NCBI’s nonredundant database (E-value ≤1e^−5^) showed homology of 2,902 genes to *L. rhamnosus* proteins. Gene Ontology (GO) analysis identified 1,020 sequences in biological processes, 1,223 in molecular functions, and 818 in cellular components. The draft genome indicates involvement in key metabolic pathways, genetic information processing, and cellular processes.

## Data Availability

The assembled de novo whole-genome sequences were deposited at GenBank (accession number JASBQR000000000.1), with general details available under BioProject identification number PRJNA966947, BioSample number SAMN34568646, and SRA project number SRR28740973.
